# Advances in research on the association between global air pollution and atopic dermatitis

**DOI:** 10.3389/fpubh.2026.1854212

**Published:** 2026-08-11

**Authors:** Boyang Xue, Yicheng Zhang, Weihao Cheng, Jing Fan, Xuesong Jia, Xue Wang

**Affiliations:** 1 Department of Dermatology, The First Affiliated Hospital of Shihezi University, Shihezi, China; 2 Medical College of Shihezi University, Shihezi, China

**Keywords:** air pollution, atopic dermatitis, gene–environment interaction, oxidative stress, particulate matter, skin barrier

## Abstract

**Background:**

Escalating global air pollution poses severe threats to public health. Beyond respiratory and cardiovascular damage, air pollutants adversely affect cutaneous health. Atopic dermatitis (AD), a common chronic inflammatory skin disease, has been closely linked to environmental pollutant exposure in recent research, arousing widespread academic concern.

**Objective:**

This narrative review aimed to comprehensively summarize current evidence on the correlation between air pollution and AD, and further elaborate the epidemiological features, potential pathogenic mechanisms, as well as targeted prevention and therapeutic strategies for pollution-related AD.

**Methods:**

Literature was retrieved from PubMed, Web of Science and CNKI databases covering April 2021 to December 2025, with core keywords related to air pollutants, AD, pathogenesis, exposure and climate change. Following narrative review guidelines, high-quality peer-reviewed studies with an impact factor ≥ 3.0 were included, focusing on cutting-edge evidence regarding air pollutant-AD associations, climatic and geographical disparities, microbiome interactions, and novel therapies.

**Results:**

Exposure to particulate matter, nitrogen dioxide, ozone and cigarette smoke significantly increases AD incidence and severity. Key mechanisms include skin barrier damage, skin microbiota dysbiosis, oxidative inflammatory stress, aryl hydrocarbon receptor pathway activation, and epigenetic gene–environment interactions. Urban residents, children, pregnant women and outdoor workers are high-risk populations. Biologics and JAK inhibitors effectively relieve AD symptoms, while antioxidants and medical ozone therapy are promising adjuvant or emerging interventions.

**Conclusion:**

Air pollution is a critical modifiable risk factor for AD onset and exacerbation via multi-pathway mechanisms. Integrated prevention, skincare and clinical treatment can reduce AD burden. Current evidence is limited to observational studies, lacking definitive causal verification. Future multicenter prospective cohort studies, standardized novel therapy application and AI-assisted precise management will facilitate systematic AD prevention and control.

## 1 Introduction

Since the turn of the 21st century, rapid global industrialization and urbanization have led to a progressive deterioration in air quality. Air pollutants not only directly affect multiple human systems, including the respiratory and cardiovascular systems, thereby inducing corresponding diseases, but may also trigger psychological problems, exerting dual adverse impacts on patients (1). Meanwhile, pollutants interact intricately with climate change, further deteriorating air quality and posing a persistent threat to public health (2). Epidemiological studies have demonstrated that the prevalence and mortality of air-pollutant-related diseases have increased markedly over the past two decades (3). As the largest human organ and the primary physical barrier against external insults, the skin is highly susceptible to air pollutants, which may thereby induce or exacerbate a spectrum of cutaneous disorders (4).

Atopic dermatitis (AD) is a common chronic inflammatory skin disease affecting approximately 129 million individuals worldwide, with a steadily rising incidence (5). Characterized by eczematous lesions, severe pruritus, and xerosis cutis, AD presents recurrent and intractable clinical manifestations (6). Its pathogenesis is multifactorial, involving the interplay of genetic susceptibility, immune dysregulation, and environmental factors. Beyond impairing cutaneous health, persistent pruritus-related sleep disturbance severely compromises patients’ overall quality of life (7).

In recent years, accumulating evidence has validated the association between air pollutants and AD. For instance, exposure levels of air pollutants are positively correlated with AD risk; a reduction in pollutant concentration can decrease AD incidence risk by approximately 15.5% (8), providing robust evidence for their causal relationship and the detrimental effects of pollutants on AD progression. Furthermore, extreme climatic events such as sandstorms and wildfires release massive amounts of particulate matter that aggravate AD severity (9), indicating synergistic pathogenic effects of climate change and air pollution in AD onset, which should be incorporated into comprehensive prevention and control strategies.

Therefore, a comprehensive elaboration of the mechanisms, epidemiological characteristics, and prevention and therapeutic strategies linking global air pollution to AD is of great significance for reducing disease burden and improving clinical outcomes among AD patients.

## 2 Research methods

### 2.1 Literature search strategy

This narrative review was conducted to systematically summarize and analyze current evidence regarding the association between air pollution and atopic dermatitis (AD). To ensure comprehensiveness and representativeness of literature retrieval, three databases were searched: China National Knowledge Infrastructure (CNKI), PubMed, and the Web of Science Core Collection, covering publications from April 2021 to December 2025, with a restriction to English-language articles. A combined search strategy of MeSH terms and free-text terms was adopted. The English search terms included “Air Pollutants,” “Particulate Matter,” “PM2.5,” “Atopic Dermatitis,” “AD,” “Pathogenesis,” “Exposure,” and “Climate Change.” Search strings were constructed using Boolean operators, with syntax adjusted according to database-specific features. In addition, reference lists of included articles were manually screened to identify potentially relevant studies and maximize retrieval coverage. The literature search was conducted by Boyang Xue, Yicheng Zhang and Weihao Cheng; study selection was performed independently by Boyang Xue and Yicheng Zhang, with disagreements resolved through discussion and final arbitration by Jing Fan, Xuesong Jia and Xue Wang.

It should be noted that, as a narrative review, this study did not follow strict inclusion or exclusion criteria or the PRISMA screening protocol for systematic reviews. Instead, literature screening and synthesis were conducted based on the following principles: (1) prioritizing high-quality original studies and representative authoritative reviews in this field; (2) focusing on studies investigating the relationships between ambient air pollutants and AD prevalence, severity, or pathogenesis; (3) highlighting cutting-edge studies on extreme climatic events, geographical and climatic disparities, temperature-microbiome interactions, and emerging therapeutic strategies.

### 2.2 Literature screening and quality assessment

Boyang Xue and Yicheng Zhang independently screened retrieved records according to the following criteria: (1) human clinical studies or animal model experiments focusing on the association between air pollutants and AD; (2) study designs including cohort studies, prospective studies, case–control studies, cross-sectional studies, and basic experimental research; (3) outcome measures covering AD incidence risk, disease severity, outpatient visit rates, and changes in relevant pathophysiological indicators.

For quality assessment, a hierarchical quality control strategy was applied: (1) journal-based screening: all included articles were peer-reviewed publications with an impact factor (IF) ≥ 3.0. IF values were obtained from the latest Journal Citation Reports (JCR) released by Clarivate Analytics and verified via the Web of Science Core Collection; (2) supplementary quality indicators: in addition to IF, studies conducted by highly cited researchers or funded by national grants were preferentially included for further validation.

**Figure 1:**
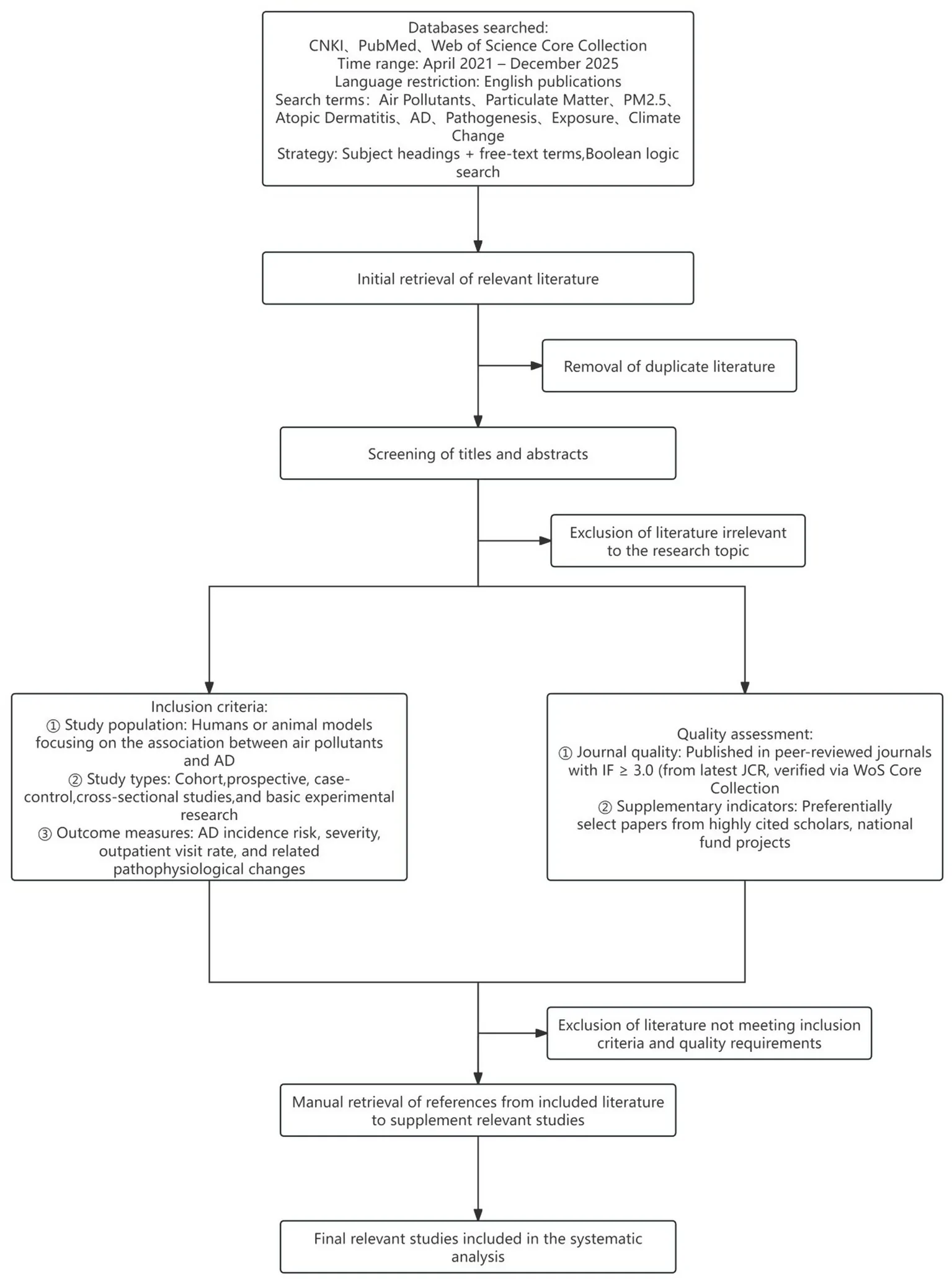
Literature retrieval and screening workflow for this narrative review.

The literature retrieval and screening workflow is illustrated in Figure 1.

Limitations: By nature, the literature screening process of a narrative review inevitably involves subjective judgments, which fundamentally differs from the objective and standardized screening procedures of systematic reviews. Although efforts were made to ensure comprehensiveness and objectivity, potential literature omission and selection bias may still exist.

## 3 Association between global air pollution and atopic dermatitis

### 3.1 Overview of global air pollutants

Air pollution refers to the deterioration of air quality caused by chemical, physical, or toxic hazardous substances suspended in the atmosphere (10). Such pollutants are mainly divided into two categories: atmospheric particulate matter and gaseous pollutants. Skin damage occurs via two primary pathways: direct cutaneous absorption of airborne pollutants, and pollutant transportation to skin tissues after entering the blood circulation through the respiratory tract (11).

Atmospheric particulate matter (PM) is a common class of air pollutants, classified by particle size into coarse particles (PM10), fine particles (PM2.5), and ultrafine particles, all of which are closely associated with the development of multiple diseases (12). Accumulating evidence supports the link between PM exposure and AD onset: increased particulate concentration correlates with higher outpatient and emergency visits for AD and with greater disease severity (13, 14). A large-sample cohort study by Chen et al. further confirmed that short-term PM2.5 exposure is strongly associated with increased healthcare utilization related to AD-related symptoms, indicating that atmospheric particulate matter constitutes a critical risk factor for inflammatory skin diseases (15).

Beyond particulate matter, gaseous pollutants represent another major category, primarily including nitrogen oxides, sulfur dioxide (SO₂), and ozone (O₃) (16), all of which also show strong correlations with AD development. A Taiwan-based study reported that nitrogen dioxide (NO₂) exposure is linked to elevated AD risk, with long-term high-concentration NO₂ exposure further increasing susceptibility (17). Moreover, adult females and children exhibit higher vulnerability to AD induced by air pollution, as evidenced by positive correlations between concentrations of SO₂, NO₂, and O₃ and outpatient visit frequencies among these populations (18, 19).

### 3.2 Pathogenic mechanisms

Based on established associations between air pollutants and AD, pollutants contribute to AD pathogenesis through multiple pathways, including skin barrier disruption, cutaneous microecological dysbiosis, induction of oxidative stress and inflammatory responses, aryl hydrocarbon receptor activation, and gene–environment interactions.

#### 3.2.1 Disruption of skin barrier function

The skin barrier consists of four interconnected layers: microbial, chemical, physical, and immune barriers, forming a highly integrated defensive network against exogenous pathogenic microorganisms (20). In AD pathogenesis, loss-of-function mutations in filaggrin (FLG), a key skin barrier protein, compromise skin integrity, increase disease risk, facilitate early-onset AD, and may lead to persistent symptoms into adulthood (21).

Air pollutants participate in AD initiation and progression by damaging the skin barrier. Roh et al. demonstrated that PM2.5 downregulates FLG mRNA expression, inducing skin barrier defects and elevating AD risk (22). PM10 also triggers or exacerbates AD-related inflammation via differential expression of skin barrier-associated protein genes (23). Transepidermal water loss (TEWL), a marker of skin barrier integrity, increases upon barrier impairment (24). Multiple pollutants, including nitrogen dioxide, cigarette smoke, volatile organic compounds, and formaldehyde, aggravate barrier damage by elevating TEWL (25).

Furthermore, geographical and climatic factors indirectly affect skin barrier function and AD risk by modulating pollutant concentrations. Both persistent high and low ambient temperatures alter the levels and distribution of air pollutants, thereby influencing AD incidence, with high-grade evidence supporting temperature-pollutant associations (26). Mechanistically, low temperature combined with low environmental humidity synergistically impairs the skin barrier: low temperatures inhibit stratum corneum lipid and water synthesis while enhancing skin permeability and reactivity to external irritants, ultimately increasing AD susceptibility (27).

#### 3.2.2 Dysbiosis of cutaneous microecology

The skin harbors complex microbial communities essential for maintaining skin barrier homeostasis. Microbial dysbiosis disrupts normal cutaneous physiology and triggers various skin disorders (28).

AD patients display distinct skin microbial composition and diversity compared with healthy individuals, characterized by reduced commensal bacteria and increased colonization rate and density of *Staphylococcus aureus*, which positively correlates with lesion severity (29). Air pollutants affect skin microbiomes in multiple ways: they reduce microbial abundance and alter community composition and function (30, 31), weaken microbial network connectivity, and lower community diversity and richness, thereby increasing cutaneous disease susceptibility (32). Pollutant-mediated cutaneous microecological dysbiosis reciprocally reinforces skin barrier injury, jointly driving AD onset and progression.

Notably, beyond direct pollutant effects, interactions between environmental factors and skin microecology also influence AD development. Individuals born in autumn or winter exhibit significantly higher AD risk and greater susceptibility to *Staphylococcus aureus* colonization, which further amplifies atopic progression (33).

#### 3.2.3 Induction of oxidative stress and inflammatory responses

Oxidative stress (OS) denotes a pathological state of imbalance between pro-oxidant and antioxidant systems characterized by excessive accumulation of reactive oxygen species (ROS). High ROS levels induce DNA and mitochondrial damage, trigger aberrant activation of cellular apoptosis and autophagy, and ultimately provoke cutaneous inflammation (34). Elevated oxidative stress-related biomarkers have been detected in AD patients (35). PM impairs endogenous antioxidant defenses, sustaining cellular OS; meanwhile, phagocytosis of PM stimulates ROS production, markedly increasing AD risk (36). In addition, combined exposure to PM and ultraviolet (UV) radiation substantially boosts intracellular ROS generation, amplifying oxidative stress effects (37). Notably, UV plays a dual role in skin inflammation: narrow-band UV alleviates AD inflammation via immunosuppression and serves as a mainstream clinical therapy (38). Nevertheless, the pathogenic effects of combined PM-UV exposure are currently inferred only at the cellular level, with clinical evidence in intact skin barriers remaining unclear and requiring prospective validation. Pollutants, including nitrogen dioxide, ozone, and sulfur dioxide, also participate in AD pathogenesis through oxidative stress pathways (39).

Apart from oxidative stress, aberrant inflammatory activation represents another core mechanism by which ROS mediate pollutant-induced AD. ROS trigger massive pro-inflammatory cytokine release, which further stimulates inflammatory cells to produce more ROS, forming a vicious cycle that exacerbates cutaneous inflammation (40). ROS also activate key transcription factors to induce expression of inflammation-related genes; moreover, particulate exposure additionally activates inflammasomes, amplifying inflammatory cascades and worsening skin injury (41). In AD mouse models, PM exposure upregulated expression of skin inflammatory biomarkers and downregulated keratinocyte differentiation markers, verifying its aggravating effects on AD progression (42).

Temperature variations also modulate AD via inflammatory pathways. Low temperatures induce pro-inflammatory cytokine production, cause epidermal barrier protein deficiency and skin barrier dysfunction, facilitate environmental toxin invasion, and further activate inflammatory responses, potentially triggering AD in healthy individuals and promoting chronicity (43). Moreover, air pollutants exert pronounced impacts on AD under specific exposure scenarios. For instance, short-term exposure to wildfire-derived air pollutants significantly increases AD outpatient visits among the older population. Researchers hypothesize a dual-mechanism pathway: wildfires, as extreme climatic events, drastically elevate particulate concentrations to directly exacerbate cutaneous oxidative stress and inflammation; meanwhile, age-related skin barrier deterioration reduces pollutant defense capacity in older adults, with synergistic effects triggering acute AD flares (44, 45).

#### 3.2.4 Activation of aryl hydrocarbon receptor

The aryl hydrocarbon receptor (AhR) is widely expressed in skin tissues and activated by diverse exogenous and endogenous ligands, playing a pivotal role in cutaneous physiological regulation. It modulates immune cell balance, cytokine secretion, skin barrier integrity maintenance, and epidermal cell differentiation (46, 47). Air pollutants are implicated in AD pathogenesis via AhR activation: polycyclic aromatic hydrocarbons carried by pollutants specifically bind to cytoplasmic AhR, sustaining persistent receptor activation (48). Such aberrant activation induces expression and secretion of multiple inflammatory factors, disrupts local cutaneous immune homeostasis, and initiates AD (49). Furthermore, activated AhR regulates neurotrophic factor expression, triggering pruritus and scratching behaviors; repetitive scratching further impairs the skin barrier and worsens AD lesions (50).

A small number of studies have challenged the role of AhR activation in AD, reporting no significant association (51). Overall, differential ligand-binding specificity and downstream signaling effects among AhR subtypes may account for inconsistent findings regarding its role in AD (52).

#### 3.2.5 Gene–environment interaction

Air pollutants modulate AD onset and progression by altering gene expression. As noted above, PM2.5 represses expression of barrier-associated proteins such as FLG via epigenetic silencing, thereby compromising skin barrier function. Additional studies have shown that PM exposure activates the pregnane X receptor signaling pathway in the skin of AD patients, with upregulated expression of downstream genes further exacerbating AD symptoms (53). Moreover, polycyclic aromatic hydrocarbons in pollutants modify human epigenetic profiles (54). Maternal air pollutant exposure during pregnancy increases AD risk in neonates, potentially mediated by pollutant-induced epigenetic modifications in mothers or fetuses (55). These findings highlight the critical role of synergistic gene–environment interactions in AD pathogenesis.

In addition, combined exposure to extreme temperatures and climate change across different life stages contributes to AD via epigenetic modifications in skin and systemic tissues (56). Cellular-level studies further confirm that wildfire smoke exposure remodels human immune responses, with such immune impairments amplified by climate change (57). Collectively, these data indicate synergistic exacerbating effects of climate change and air pollution on AD development. Geoclimatic determinants, including humidity, temperature, and pollution events, modulate pollutant exposure patterns and cutaneous immune microenvironments, thereby governing AD flare severity and frequency.

**Figure 2:**
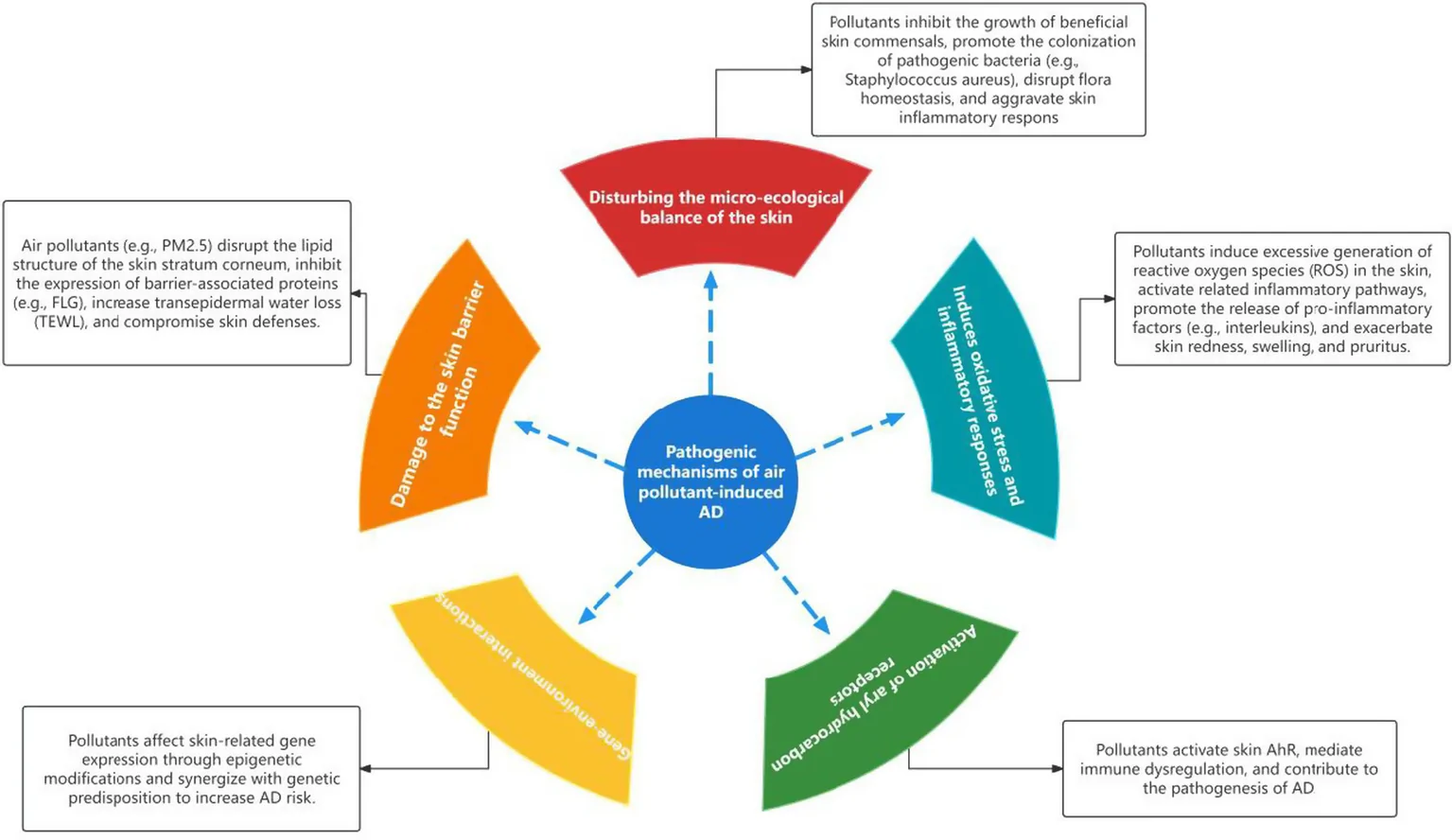
Pathogenic mechanisms of air pollutant-induced atopic dermatitis: a multifaceted overview.

The pathogenic mechanisms of the above pollutants are systematically visualized in Figure 2, which intuitively displays the multi-pathway synergistic action mode of air pollutants in inducing and aggravating AD, reflecting the interconnection and mutual promotion among each pathogenic link. The specific action mechanisms of different types of air pollutants and their involved AD pathogenic links are summarized in Table 1.

**Table 1:** Mechanisms of atopic dermatitis (AD) induced by different air pollutants.

Pollutant type	Primary target pathways	Distinctive molecular/Cellular events
Particulate matter (PM)	Skin barrier; Oxidative stress; Inflammation; AhR signaling; Gene regulation	Epigenetic silencing of FLG via promoter methylation Pregnane X receptor (PXR) pathway activation with downstream gene upregulation Cellular phagocytosis of PM particles as ROS generation mechanism PAH-specific AhR ligand-receptor binding with subtype-dependent divergent effects
Nitrogen dioxide (NO₂)	Skin barrier; Oxidative stress; Inflammation	TEWL elevation as specific barrier integrity biomarker Long-term cumulative exposure dose–response relationship Gender- and age-specific susceptibility (adult females, children)
Ozone (O₃)	Oxidative stress; Inflammation	ROS accumulation without particulate phagocytosis requirement Direct lipid peroxidation of stratum corneum lipids
Sulfur dioxide (SO₂)	Oxidative stress; Inflammation	Acidic stress mechanism (sulfite/sulfate radicals) Interaction with airborne particles to form secondary PM

Pollutant-specific mechanisms in AD. PM, particulate matter; FLG, filaggrin; PXR, pregnane X receptor; ROS, reactive oxygen species; PAH, polycyclic aromatic hydrocarbon; AhR, aryl hydrocarbon receptor; TEWL, transepidermal water loss.

### 3.3 Epidemiological characteristics

#### 3.3.1 Regional differences

Due to variations in industrialization level, urbanization progress, geographical and climatic features, and air pollution severity across regions, the impacts of air pollution on atopic dermatitis (AD) exhibit pronounced regional heterogeneity. Epidemiological data demonstrate uneven global prevalence of AD, ranging from 1.62% in Central Asia to 4.05% in central sub-Saharan Africa. However, AD-related research is relatively scarce in South Africa, South America, and Oceania, resulting in limited representativeness of available data in these areas (58).

Regional disparities also exist in the pathogenic contribution of AD-susceptible genes. The filaggrin (FLG) gene mutation mentioned above exerts variable pathogenic effects across populations: it accounts for 50% of AD cases in European populations and 27% in Asian populations, with a prevalence of 7–10% among Caucasian Europeans (59). Such regional differences in genetic background further modify population susceptibility to air pollutants, thereby exacerbating geographical heterogeneity in AD prevalence.

Collectively, existing evidence indicates a higher AD prevalence in developed countries, which may be closely attributed to aggravated air pollution driven by rapid urbanization.

#### 3.3.2 Exposure-related differences and susceptible populations

AD risk is closely associated with air pollutant exposure. A pediatric AD study reported a higher prevalence among urban children compared with suburban children with lower pollutant exposure; children living in households with regular or occasional smokers also showed increased AD risk relative to those in non-smoking households (60).

Exposure patterns among vulnerable subgroups warrant particular attention. First, prenatal particulate matter exposure has been confirmed to increase childhood AD risk. Further evidence indicates that maternal prenatal exposure to other harmful air pollutants, such as cigarette smoke, also elevates AD susceptibility in offspring (61). Second, outdoor workers, including traffic police and farmers, are continuously exposed to ambient air pollutants, with persistent cutaneous stimulation potentially promoting the onset and progression of AD and other inflammatory skin disorders.

To facilitate clinical and public health application, we summarize key studies from this review investigating the association between air pollution, climatic factors, and AD severity in Table 2.

**Table 2:** Key studies on the association between air pollution, climatic factors, and atopic dermatitis (AD) severity.

Year	Study (First Author)	Environmental variables	Main findings
2021	Roh et al. (22)	PM2.5	PM2.5 downregulates FLG mRNA expression, inducing skin barrier defects and elevating AD risk
2021	Brown et al. (58)	Regional air pollution, industrialization level	AD prevalence uneven globally (1.62% Central Asia to 4.05% central sub-Saharan Africa); higher prevalence in developed countries attributed to rapid urbanization and severe air pollution
2022	Lee et al. (17)	NO₂, long-term high-concentration exposure	NO₂ exposure linked to elevated AD risk; long-term high-concentration NO₂ exposure further increases susceptibility
2022	Hwang et al. (18, 19)	SO₂, NO₂, O₃	Adult females and children exhibit higher vulnerability; positive correlations between concentrations of SO₂, NO₂, and O₃ and outpatient visit frequencies
2022	Wang et al. (60)	Urban vs. suburban PM, household smoking	Higher prevalence among urban children vs. suburban children; children in regular or occasional smoking households showed increased AD risk
2022	Zhang et al. (33)	Seasonal birth (autumn/winter), *S. aureus* colonization	Individuals born in autumn or winter exhibit significantly higher AD risk and greater *S. aureus* colonization susceptibility
2023	Chen et al. (15)	PM2.5	Short-term PM2.5 exposure strongly associated with increased healthcare utilization related to AD symptoms; PM constitutes critical risk factor for inflammatory skin diseases
2023	Kim et al. (44, 45)	Wildfire-derived air pollutants, extreme climatic events	Short-term wildfire pollutant exposure significantly increased AD outpatient visits among older population; drastic elevation of particulate concentrations directly exacerbates cutaneous oxidative stress and inflammation; age-related barrier deterioration reduces pollutant defense capacity
2023	Li et al. (26, 27)	Temperature, humidity, PM	Both persistent high and low ambient temperatures alter pollutant levels and distribution, influencing AD incidence; low temperature combined with low humidity synergistically impairs skin barrier
2023	Park et al. (36, 37)	PM, UV radiation	Combined PM-UV exposure substantially boosts intracellular ROS generation; pathogenic effects inferred at cellular level, clinical evidence in intact skin barriers unclear

Key studies on the association between air pollution, climatic factors, and atopic dermatitis (AD) severity, arranged chronologically by publication year. PM, particulate matter; FLG, filaggrin; NO₂, nitrogen dioxide; SO₂, sulfur dioxide; O₃, ozone; UV, ultraviolet; ROS, reactive oxygen species; *S. aureus*, *Staphylococcus aureus*.

## 4 Prevention and treatment strategies

### 4.1 Prevention and control of air pollution

Targeted preventive measures are critical to mitigating the adverse impacts of air pollutants on cutaneous health and represent key steps in reducing the risk of atopic dermatitis (AD). First, enhanced environmental monitoring and comprehensive governance are required. Establishing a scientific air pollutant monitoring and analysis system enables real-time tracking of air pollution dynamics and evidence-based management strategies, thereby minimizing pollutant exposure at the source. Second, public awareness regarding the association between air pollution and skin health, particularly AD onset, should be improved. Popular science articles, videos, and public welfare lectures can disseminate mechanisms underlying pollution-induced AD and strengthen proactive self-protection awareness, laying a cognitive foundation for disease prevention. Finally, daily skin care serves as the primary defense against pollution-related damage. Regular skin cleansing to remove pollutants and barrier protection can effectively alleviate cutaneous irritation and reduce AD risk (62). Rational skin care not only prevents pollution-associated skin disorders but also facilitates long-term management of chronic skin diseases such as AD.

Furthermore, given the complex interplay between climatic conditions and air pollution—where climate change alters pollutant sources, concentrations, distribution, and seasonal patterns—prevention strategies should integrate climate adaptation with air pollution governance. A multidimensional environmental monitoring network and early-warning systems for high-risk populations should be established accordingly (63).

### 4.2 Clinical management of AD

Remarkable advances have been achieved in AD treatment in recent years. Considering that air pollutants act as major environmental drivers of AD onset, clinical management should balance symptom control and reduction of pollution-related triggers to deliver individualized comprehensive therapy.

A panel of approved effective and safe topical and systemic therapeutic options is currently available (64). Beyond conventional pharmacotherapy, biologics have emerged as a research hotspot. They effectively modulate disease-related immune responses, suppress pollutant-induced inflammation, relieve clinical manifestations, and improve patients’ quality of life (65). Dupilumab is widely used in clinical practice; abundant clinical data have demonstrated its remarkable efficacy and favorable safety profile in controlling moderate-to-severe AD inflammation and alleviating pollution-associated cutaneous symptoms (66).

Compared with biologics, small-molecule drugs penetrate cell membranes to target intracellular signaling pathways (67), conferring particular advantages in treating severe AD patients and those with long-term air pollutant exposure. Janus kinase (JAK) inhibitors such as abrocitinib are the major small-molecule agents under clinical application and investigation. Clinical evidence shows that abrocitinib exhibits potent efficacy and acceptable safety in severe AD, offering novel therapeutic options for AD patients chronically affected by air pollution (68).

Notably, ambient ozone (O₃), a harmful air pollutant that triggers oxidative stress and exacerbates skin injury, is fundamentally distinct from therapeutic ozone, which must be clearly differentiated to avoid patient confusion. Ambient ozone pollution disrupts the skin barrier and induces inflammatory responses, thereby worsening AD (69); by contrast, therapeutic ozone specifically ameliorates cutaneous microecological dysbiosis and aberrant local inflammation in AD patients. Based on this property, studies have explored its application in AD treatment, showing that topical ozone may modulate local immune responses and restore skin microbiota, providing a promising therapeutic direction (70).

For adjuvant therapy, antioxidant supplementation maintains reactive oxygen species (ROS) homeostasis and improves AD outcomes (71). Researchers have proposed incorporating antioxidant strategies into comprehensive AD management: vitamin supplementation, melatonin intake, balanced diets, smoking avoidance, and stress reduction alleviate oxidative stress and lower recurrence risk (72). As a potent antioxidant, melatonin plays a vital role in AD pathogenesis; low serum melatonin levels correlate with AD inflammation. Melatonin neutralizes free radicals, regulates antioxidant enzyme activity, inhibits inflammasome activation, and systematically relieves cutaneous inflammation. Moreover, melatonin metabolites exert antioxidant and anti-inflammatory effects, amplifying and prolonging its biological functions (73).

**Figure 3:**
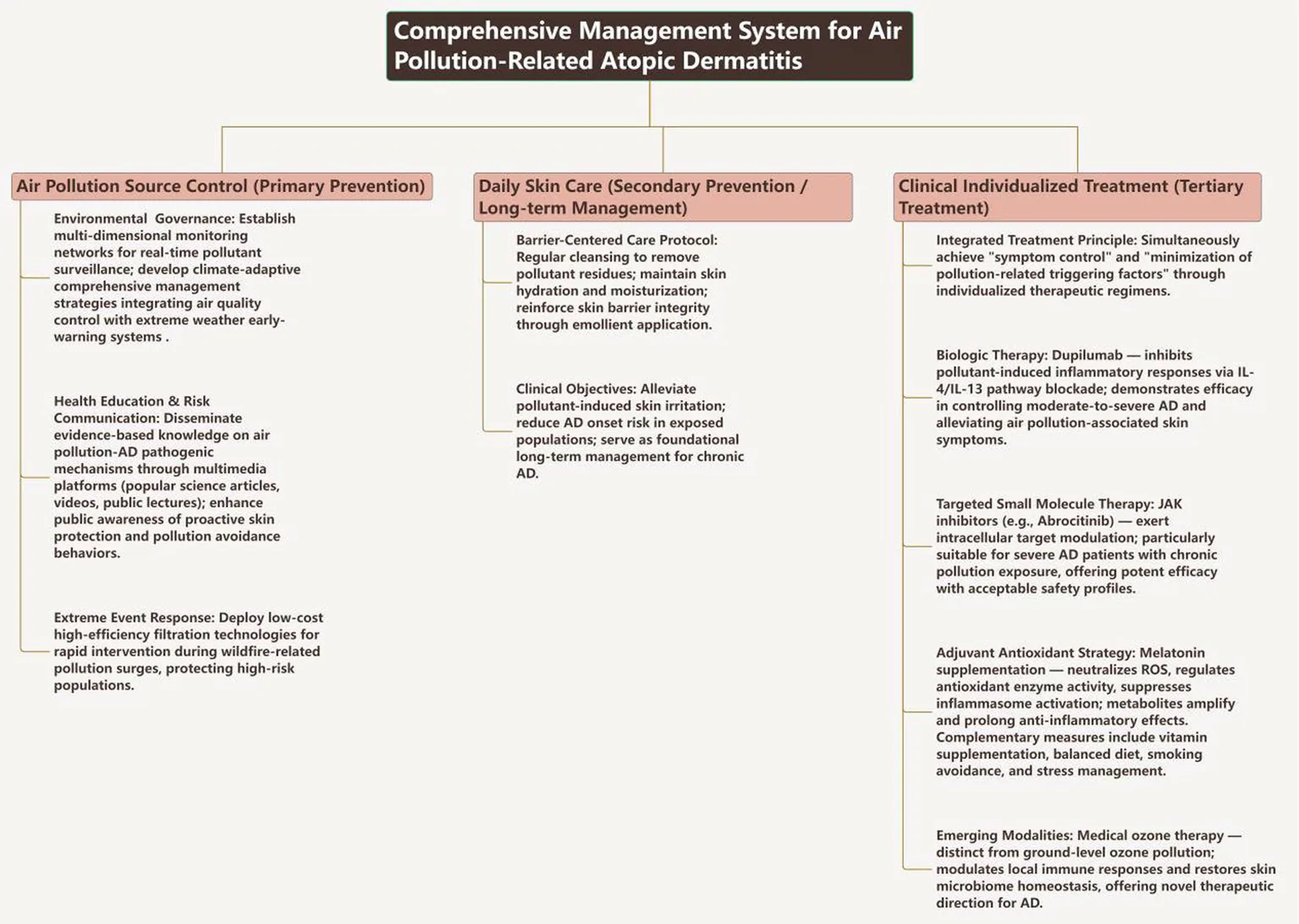
Integrated response system for air pollution-related atopic dermatitis.

For the prevention and treatment of pollution-related AD, we have constructed a multi-level integrated response system, which is clearly presented in Figure 3.

## 5 Summary and outlook

Air pollutant exposure has emerged as a critical public health issue requiring widespread attention, with its detrimental effects on cutaneous health becoming increasingly prominent. In particular, air pollution acts as a key environmental driver in the onset and progression of atopic dermatitis (AD). Using a narrative review approach, this paper comprehensively summarizes the association between air pollutants and AD, systematically elaborates multidimensional pathogenic pathways by which pollutants induce or exacerbate AD, describes epidemiological distribution patterns of AD, and proposes integrated prevention and treatment strategies.

In terms of pathogenic mechanisms, pollutants contribute to AD not through a single pathway, but via an interconnected and mutually amplifying network of five major mechanisms: skin barrier disruption, cutaneous microecological dysbiosis, induction of oxidative stress and inflammatory responses, aryl hydrocarbon receptor (AhR) activation, and gene–environment interactions. These pathways do not function independently; instead, they form a synergistic amplification network modulated by geoclimatic factors, including temperature, humidity, and extreme pollution events. Epidemiologically, AD exhibits marked regional heterogeneity and population-specific susceptibility. Developed countries generally show higher AD prevalence owing to rapid urbanization and severe air pollution. High-risk populations requiring prioritized protection include children, pregnant women, and long-term outdoor workers such as traffic police and farmers, due to physiological vulnerability or frequent pollutant exposure. Regarding prevention and management, air pollution control reduces AD triggers through environmental and climate governance, public health education, and daily skin care. Clinically, in addition to conventional medications, biologics such as dupilumab and Janus kinase (JAK) inhibitors such as abrocitinib have become core therapeutic agents that specifically alleviate pollution-associated AD manifestations. Given the pivotal role of oxidative stress in pollutant-mediated AD, antioxidants such as melatonin can serve as adjuvant therapies. Medical ozone therapy, an emerging intervention fundamentally distinct from ambient ozone pollution, shows promising potential; nevertheless, further validation of its safety and efficacy is warranted.

**Figure 4:**
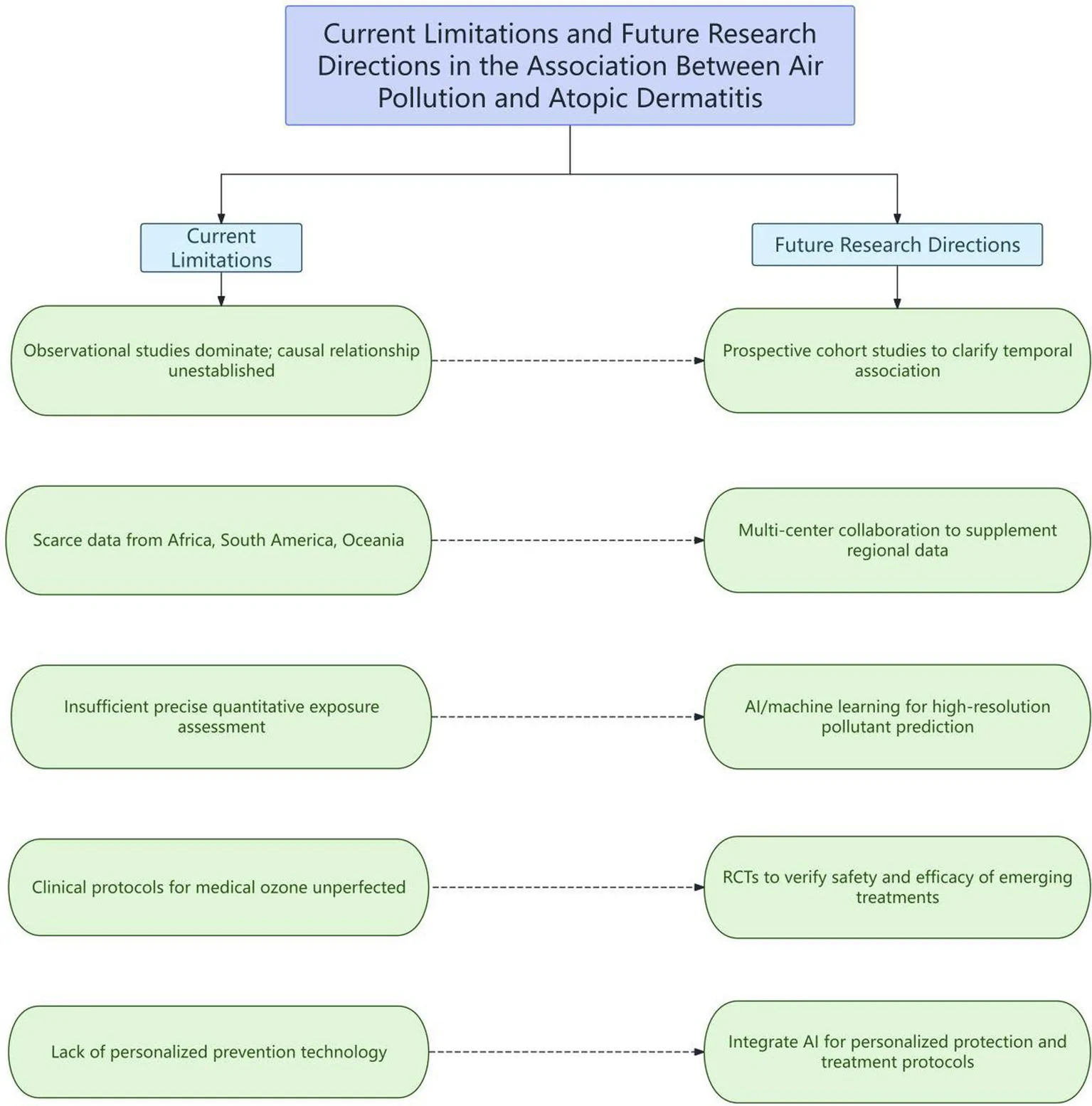
Current limitations and future research directions in the association between air pollution and atopic dermatitis (AD).

Nevertheless, several important limitations of this review must be acknowledged, and corresponding future research directions should be pursued to address these gaps in Figure 4. First, limitations in causal inference remain. Most existing evidence originates from observational and epidemiological studies, which are inherently prone to selection bias, information bias, and confounding factors, making it difficult to establish definitive causality between pollutant exposure and AD onset. Second, regional representativeness is insufficient. Relevant studies are scarce in regions including Africa, South America, and Oceania, resulting in limited data generalizability worldwide. Third, validation of therapeutic interventions remains inadequate. Clinical protocols for emerging therapies such as medical ozone remain underdeveloped; their long-term safety, optimal dosage regimens, and clear differentiation from ambient ozone pollution require further verification. The clinical significance of oxidative stress in AD, particularly the efficacy of antioxidant-based therapies, also needs confirmation via randomized controlled trials. Finally, personalized prevention technologies are lacking. Precision prediction models based on individual genetic background, exposure history, and skin status have not yet been established, and the complex synergistic effects of ultraviolet radiation and pollutants remain to be further elucidated.

Future research should address these limitations to promote high-quality advances in this field. On one hand, more prospective cohort studies should be conducted to clarify the association between AD and air pollutants. Randomized controlled trials on medical ozone therapy are needed to define its indications, administration regimens, and long-term safety, while cost-effectiveness assessments of targeted antioxidant therapies will help establish clinical guidelines. On the other hand, artificial intelligence (AI) technologies should be harnessed. Research teams have successfully constructed machine-learning models to predict and estimate key environmental indicators such as long-term average particulate number concentration and mean particle size at a high spatial resolution of 5 m (74). Meanwhile, machine-learning models have assisted in identifying therapeutic biomarkers for AD (75). Such technological breakthroughs not only fill technical gaps in research on air pollution and skin health but also provide critical approaches to overcoming traditional diagnostic and therapeutic bottlenecks and refining personalized management strategies. Future work should integrate pollutant exposure monitoring, mechanistic investigation, and precision therapeutics to develop more comprehensive and efficient prevention and management protocols for pollution-related skin diseases, ultimately advancing AD diagnosis and treatment toward precision, personalization, and systematization.
